# Long-Term Clinical Outcome and Carrier Phenotype in Autosomal Recessive Hypophosphatemia Caused by a Novel *DMP1* Mutation

**DOI:** 10.1002/jbmr.105

**Published:** 2010-04-07

**Authors:** Outi Mäkitie, Renata C Pereira, Ilkka Kaitila, Serap Turan, Murat Bastepe, Tero Laine, Heikki Kröger, William G Cole, Harald Jüppner

**Affiliations:** 1 Hospital for Children and Adolescents, University of Helsinki Helsinki, Finland; 2 Folkhälsan Institute of Genetics, Biomedicum Helsinki Helsinki, Finland; 3 Department of Pediatrics, David Geffen School of Medicine, University of California Los Angeles Los Angeles, CA, USA; 4 Endocrine Unit, Massachusetts General Hospital and Harvard Medical School Boston, MA, USA; 5 Department of Surgery, Bone and Cartilage Research Unit, Kuopio University Hospital Kuopio, Finland; 6 Division of Orthopaedic Surgery, The Hospital for Sick Children, University of Toronto Toronto, Ontario, Canada

**Keywords:** hypophosphatemia, rickets, osteomalacia, skeletal dysplasia, DMP1, FGF-23

## Abstract

Homozygous inactivating mutations in *DMP1* (*dentin matrix protein 1*), the gene encoding a noncollagenous bone matrix protein expressed in osteoblasts and osteocytes, cause autosomal recessive hypophosphatemia (ARHP). Herein we describe a family with ARHP owing to a novel homozygous *DMP1* mutation and provide a detailed description of the associated skeletal dysplasia and carrier phenotype. The two adult patients with ARHP, a 78-year-old man and his 66-year-old sister, have suffered from bone pain and lower extremity varus deformities since early childhood. With increasing age, both patients developed severe joint pain, contractures, and complete immobilization of the spine. Radiographs showed short and deformed long bones, significant cranial hyperostosis, enthesopathies, and calcifications of the paraspinal ligaments. Biochemistries were consistent with hypophosphatemia owing to renal phosphate wasting; markers of bone turnover and serum fibroblast growth factor 23 (FGF-23) levels were increased significantly. Nucleotide sequence analysis of *DMP1* revealed a novel homozygous mutation at the splice acceptor junction of exon 6 (IVS5-1G > A). Two heterozygous carriers of the mutation also showed mild hypophosphatemia, and bone biopsy in one of these individuals showed focal areas of osteomalacia. In bone, DMP1 expression was absent in the homozygote but normal in the heterozygote, whereas FGF-23 expression was increased in both subjects but higher in the ARHP patient. The clinical and laboratory observations in this family confirm that DMP1 has an important role in normal skeletal development and mineral homeostasis. The skeletal phenotype in ARHP may be significantly more severe than in other forms of hypophosphatemic rickets. © 2010 American Society for Bone and Mineral Research.

## Introduction

Hypophosphatemia owing to renal phosphate wasting is inherited most frequently as an X-linked dominant disorder (XLH), which is caused by dominant mutations in the *PHEX* gene, or as an autosomal dominant condition (ADHR), which is caused by heterozygous mutations in the fibroblast growth factor 23 gene (*FGF23*).(1) Recently, homozygous mutations in the gene encoding DMP1 (dentin matrix protein 1), a noncollagenous phosphoprotein, were identified as the cause of an autosomal recessive form of hypophosphatemia (ARHP).(2,3)

*Dmp1* was identified originally from a rat incisor cDNA library and was shown to play an important role in late-stage dentinogenesis.(4) Further studies have shown that DMP1 is expressed mainly in mineralizing tissues.(5,6) Dmp1-deficient mice have no apparent abnormalities in prenatal bone development. However, postnatal growth plate development is severely impaired and results in a chondrodysplasia-like phenotype characterized by short and widened long bones with flaired and irregular metaphyses and delayed and malformed ossification centers.(3,7) Microscopically, the growth plates are expanded and disorganized with increased proliferation of chondrocytes, impaired chondrocyte apoptosis, poor calcification of cartilage matrix, and delayed blood vessel invasion.(3,7) The skeletal abnormalities in *Dmp1*-null mice are 100% penetrant and worsen with age, whereas heterozygous mutation carriers show no skeletal abnormalities.(7)

Ling and colleagues(8) showed that *Dmp1*-null mice have a decreased mineral-to-matrix ratio in bones and that DMP1 has both direct and indirect roles in the regulation of postnatal skeletal mineralization. More importantly, *Dmp1*-null animals revealed significantly lower serum phosphorus concentrations than wild-type controls owing to increased urinary phosphate excretion, which is associated with increased FGF-23 levels.(9) Subsequent studies showed that the hypophosphatemia and bone abnormalities in *Dmp1*-null animals can be normalized by the transgenic expression of the full-length or the C-terminal 57-kDa fragment of DMP1 under the control of the DMP1 promoter, suggesting that this osteocyte/osteoblast-derived protein has a major role in reducing FGF-23 synthesis and/or secretion.(10) Consistent with these findings in mice, Feng and colleagues(3) and Lorenz-Depiereux and colleagues(2) recently described several unrelated families with autosomal recessive hypophosphatemia caused by homozygous loss-of-function mutations in *DMP1*. The affected individuals had clinical findings consistent with rickets/osteomalacia and metabolic profiles consistent with hypophosphatemia owing to renal phosphate wasting, normal urinary calcium excretion, normal parathyroid function, and levels of 1,25-dihydroxyvitamin D [1,25(OH)_2_D_3_] that were inappropriately normal for the degree of hypophosphatemia. They also had increased or inappropriately normal serum FGF-23 concentrations.(2,3) Bone biopsy in an affected individual showed, in addition to severe osteomalacia, increased bone volume.(3)

We now describe a Finnish family with two adults affected by ARHP owing to a novel homozygous *DMP1* mutation. The homozygous patients had a severe phenotype with skeletal dysplasia, whereas the heterozygous individuals apparently were healthy but showed, on careful assessment, a mild carrier phenotype.

## Patients and Methods

### Clinical data

The family with autosomal recessive hypophosphatemic rickets was identified through the Finnish Skeletal Dysplasia Registry. The two affected adult siblings and two clinically unaffected children, both obligate mutation carriers, were invited to participate. The Helsinki University Hospital Ethics Review Board approved the protocol, and written informed consent was obtained from all participants.

Clinical data were collected from medical records. Patients were assessed clinically, and blood and urine samples were obtained for measurement of different parameters of calcium (Ca) and phosphorus (Pi) homeostasis and of bone metabolism.

Plasma concentrations of P_*i*_, Ca, and alkaline phosphatase (ALP) and urine concentrations of P_*i*_, Ca, and creatinine (Crea) were determined by standard assays. Serum parathyroid hormone (PTH) was measured by solid-phase enzyme-labeled chemiluminescent immunometric assay (IMMULITE 2000, DPD, Diagnostic Products Corporation, Los Angeles, CA, USA). Serum 1,25(OH)_2_D_3_ was determined by RIA (Gamma-B 1,25-Dihydroxy Vitamin D RIA, Immunodiagnostic Systems, Boldon, UK) and serum 25-hydroxyvitamin D [25(OH)D] was determined by high-performance liquid chromatography.

Serum concentrations of N-terminal propeptide of type I procollagen (PINP), a bone-formation marker, and C-terminal telopeptide of type I collagen (ICTP), a bone-resorption marker, were measured by competitive RIA (UniQ PINP/ICTP RIA, Orion Diagnostica, Espoo, Finland). The second fasting morning void was collected for measurement of the bone-resorption marker urinary N-terminal telopeptide of type I collagen (U-NTX). Analyses were performed with a luminoimmunologic assay (Vitros ECi, NTx Reagent Pack, Ortho-Clinical Diagnostics, New York, NY, USA), and the results were expressed as nanomoles of bone collagen equivalents (BCEs) per millimole of creatinine. FGF-23 was measured by a two-site immunometric assay (Immutopics, San Capistrano, CA, USA) that uses antibodies directed against two C-terminal epitopes of human FGF-23 and thus detects intact FGF-23 as well as a C-terminal fragment thereof.(11)

### Radiologic evaluation

Previously obtained radiographs and other available imaging studies were reviewed. A full radiographic skeletal survey was obtained for the two affected adults, and a partial skeletal survey was obtained for the two obligate carriers. Bone mineral density (BMD) was measured with dual-energy X-ray absorptiometry (DXA, Hologic Discovery A, Hologic, Bedford, MA, USA), and the results were compared with age- and sex-specific normative data for the equipment.

### Bone histomorphometry

A transilial bone biopsy was obtained from one subject, an obligate carrier (individual 3), with a bone biopsy needle with an inner diameter of 7.5 mm (Rochester Bone Biopsy, Medical Innovations International, Inc., Rochester, MN, USA) following two courses with an oral tetracycline, which were separated by a 10-day period. The biopsy was performed 4 days after the last tetracycline administration. In addition, a previously obtained transilial bone biopsy of a female patient with ARHP (patient 1) was reanalyzed. Bone histomorphometric analysis was conducted in the Bone Histomorphometry Laboratory at UCLA. All parameters were analyzed under ×200 magnification using the OsteoMetrics system (OsteoMetrics, Decatur, GA, USA), and the results were compared with normative data.(12) The samples were stained with toluidine blue stain (pH 7.4). Mineralized bone was defined by dark-blue areas; pale-blue seams of at least 1.5 µm in width were included in the osteoid measurements. For dynamic histomorphometric analysis, mineralizing surface per bone surface and mineral apposition rate were measured in unstained sections under ultraviolet (UV) light using a B-2A set longpass filter consisting of an excitation filter ranging from 450 to 490 nm, a barrier filter at 515 nm, and a dichroic mirror at 500 nm. Nomenclature as well as all abbreviations and standard formulas follow the recommendations by the American Society for Bone and Mineral Research.(13)

### Immunohistochemistry of bone

The technique for the immunohistochemical detection of FGF-23 and DMP1 in partially decalcified bone was adapted from a previously reported method.(14,15) In brief, two adjacent 5-µm sections of bone tissue were placed side by side on each slide. Sections were deplastified in xylene and chloroform, rehydrated in graded alcohol solutions, and partially decalcified in 1% acetic acid. Endogenous peroxidase activity was quenched in 3% hydrogen peroxide–methanol solution. Nonspecific binding was blocked by an avidin-biotin solution and 5% normal horse serum with 1% bovine serum albumin. Sections then were incubated with either affinity-purified polyclonal goat anti-human FGF-23 (raised against amino acids 225 to 244; Immutopics) or monoclonal anti-human DMP1 (LFMb31) (raised against amino acids 62 to 513; kindly provided by Dr Larry Fisher, NIH) primary antibody overnight at 4°C in a humidified chamber. Subsequently, samples were incubated with specific biotinylated secondary antibody (Vector, Burlingame, CA, USA) followed by ABComplex/HRP complex (ABC Kit, Vector) and developed using the AEC Kit (Vector). Sections then were counterstained with Mayer hematoxylin (Sigma-Aldrich, St. Louis, MO, USA). Iliac crest bone biopsy specimens from four adolescent or young-adult subjects with normal renal function served as controls. Negative controls were performed for each bone section by omitting the primary antibody. The specimen sections were batched; thus immunohistochemistry was performed simultaneously on all patient specimens, along with normal and negative controls.

### Genetic studies

DNA was extracted by standard methods from peripheral blood lymphocytes.(16) Nucleotide sequence analysis of the PCR-amplified exons encoding DMP1 and the flanking intronic regions were performed at the Harvard-Partners Center for Genetics and Genomics (Boston, MA, USA). To confirm the presence of the identified mutation, *DMP1* exon 6 was amplified by using 5'-CGGTTCCTGGAATACTGACC-3' as the forward primer and 5'-GGAGTTTCCCCTTTCACTCC-3' as the reverse primer to obtain a 551-bp PCR product, which then was incubated with the endonuclease *Bfa*I. Genomic DNA from 74 healthy individuals served as control.

## Results

### Clinical characteristics

**Fig. 1:**
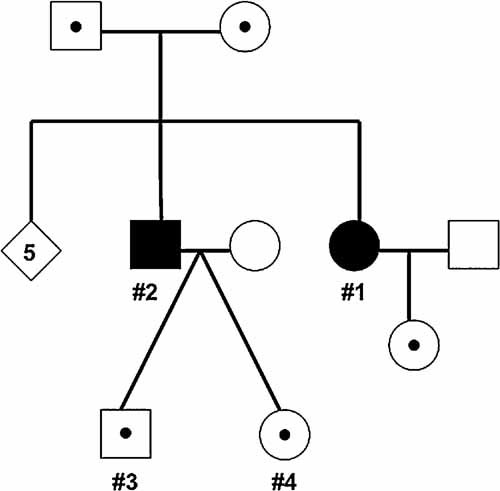
Pedigree of the family with ARHP. Black symbols = affected individuals; white symbols with black dots = carriers of the mutation.

**Fig. 2:**
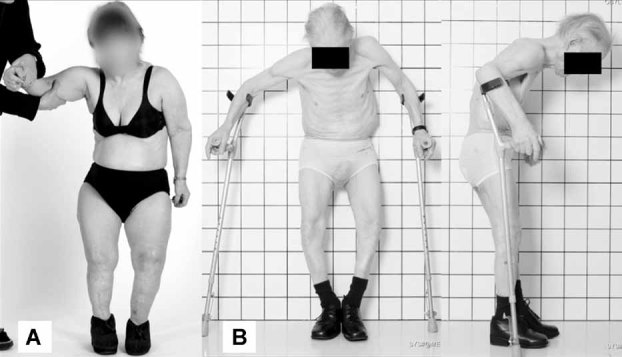
Photographs of the two adult ARHP patients. (*A*) Patient 1 at the age of 66 years. (*B*) Patient 2 at the age of 78 years.

The pedigree of the family is shown in Fig. 1. The proband, patient 1, a 66-year-old woman (Fig. 2*A*) was born to healthy parents with no known consanguinity, but both parents originated from the same remote island in southeastern Finland; she has five healthy siblings and one affected brother (patient 2). This patient has had bone pain and knee varus deformities since early childhood. Lower limb osteotomies were first performed at age 6 years, and subsequently, numerous (>30) orthopedic procedures were needed to correct lower limb deformities, to repair bilateral femoral neck fractures, and to treat cervical spinal stenosis. Total-hip replacements were performed at 57 and 61 years, respectively. The patient had significant dental problems, with recurrent gingival abscess formation, and required a dental prosthesis since age 50. She has a hearing deficit. Hypophosphatemia was diagnosed at the age of 20 years, and she was treated periodically with oral phosphate and active vitamin D analogues. Her height, when assessed for this study, was 130 cm. She has complete spinal ankylosis with calcification of the spinal ligaments and dural ectasia, lower limb deformities, and contractures.

Patient 2, a 78-year-old man (Fig. 2*B*), developed bone pain and knee varus deformity in early childhood and was first evaluated at age 3 years. Lower limb osteotomies were performed at 18 years, but hypophosphatemia was not diagnosed until adulthood, and he never received treatment with oral phosphate or vitamin D analogues. With increasing age, he developed severe joint pain and contractures, calcifications of the paraspinal ligaments leading to complete immobilization of the spine, and significant cranial hyperostosis. He has a hearing deficit and significant dental problems related to abscesses. His maximum adult height was 150 cm. When assessed for this study, he measured 138 cm in height and was wheelchair-bound. Other illnesses included hypertension and prostate cancer, diagnosed at age 66.

Individuals 3 and 4 are healthy 38-year-old adults, the twin children of patient 2, who thus are heterozygous carriers of the identified *DMP1* mutation; both had no complaints, no skeletal abnormalities, and no history of other medical problems.

### Genetic findings

Molecular genetic analysis of the *DMP1* gene revealed a novel mutation at the splice acceptor junction of exon 6 (IVS5-1G > A). Patients 1 and 2 are homozygous and individuals 3 and 4 are heterozygous carriers of the mutation. The nucleotide change, which was absent in 148 control chromosomes, is predicted to alter pre-mRNA splicing. Several potential downstream cryptic splice acceptor sites were identified that are likely to result in a shift in the open-reading frame if the resulting message is stable.

### Biochemical findings

Biochemical findings of the four assessed subjects are presented in Table 1. For both patients with the homozygous *DMP1* mutations, blood and urine biochemistries were consistent with hypophosphatemia owing to renal phosphate wasting. Markers of bone turnover were increased significantly, and 1,25(OH)_2_D_3_ concentration was inappropriately low for the degree of hypophosphatemia. Serum FGF-23 was increased in both patients. The heterozygous carriers of the mutation had mild hypophosphatemia and increased urinary phosphate excretion, whereas other biochemistries were normal (Table 1).

**Table 1:** Biochemical and Bone Densitometry Findings in the Subjects With Homozygous or Heterozygous *DMP1* Mutations

Parameter	Subject 1	Subject 2	Subject 3	Subject 4	Normal
Gender/age (years)	F/66	M/78	M/38	F/38	
Genotype	Homozygous	Homozygous	Heterozygous	Heterozygous	
Height (cm)	130	138	173	167	
Ca (mmol/L)	2.21	2.25	2.36	2.15	2.15–2.51
P_*i*_ (mmol/L)	0.60	0.48	0.48	0.70	0.71–1.23
ALP (U/L)	**191**	**290**	56	37	35–105
P-PTH (ng/L)	**89**	**93**	69	61	8–73
25(OH)D (nmol/L)	23	38	58	86	>40
1,25(OH)_2_D_3_ (pmol/L)	77	98	**138**	105	48–110
TmP/GFR	0.47	0.42	0.44	0.60	>0.80
U-INTP (nmol/mmol Cr)	**276**	**261**	28	43	<65
PINP (µg/L)	**156**	84	37	36	19–84
ICTP (µg/L)	**10.2**	**11.1**	1.8	3.1	1.5–5.0
FGF-23 (RU/ml)	305	182	53	41	<150
Lumbar spine BMD *Z*-score	NA	NA	−1.5	−0.5	>–1.0
Femoral neck BMD *Z*-score	NA	NA	−1.3	−0.7	>–1.0

*Note:* Supranormal values are in bold; subnormal values are underlined.

### Radiologic evaluation

**Fig. 3:**
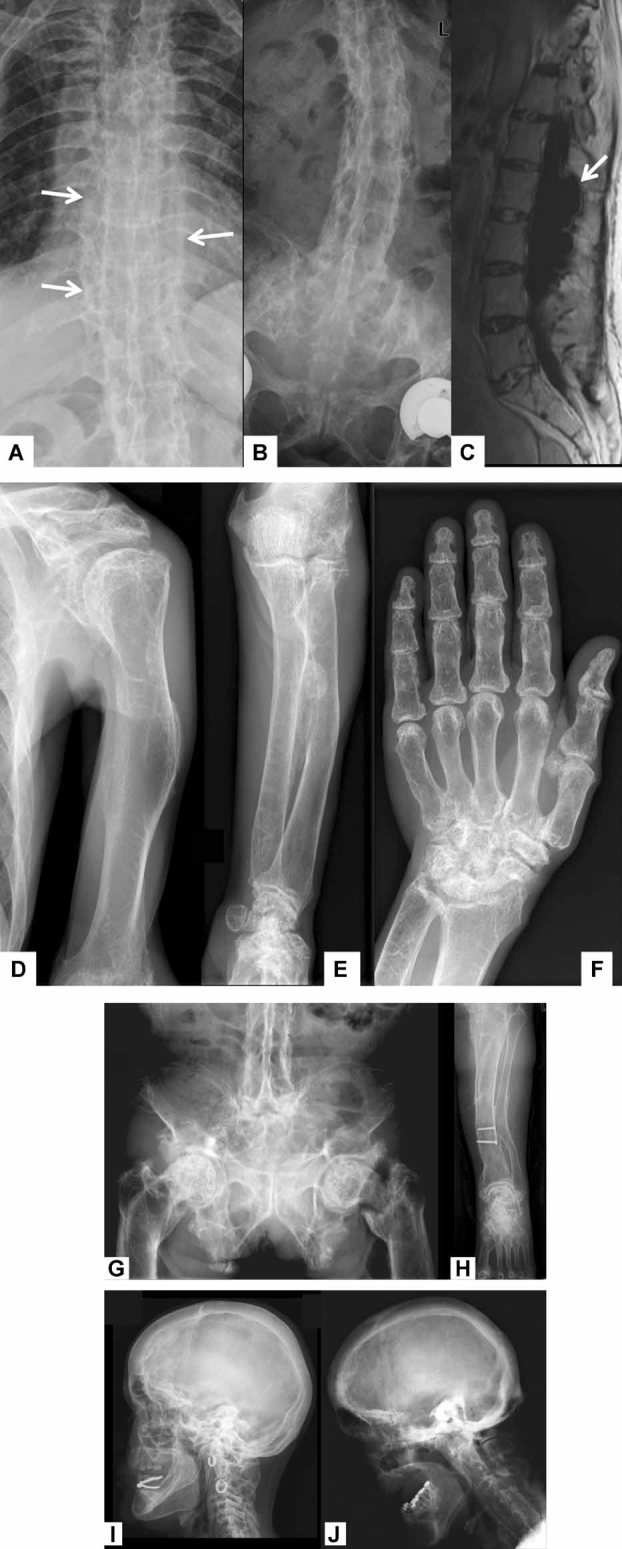
Radiographic findings in the two ARHP patients with the homozygous *DMP1* mutation. Calcifications (*arrows*) of the spinal ligaments in the thoracic (*A*) and lumbar spine (*B*) in patient 1. Spinal MRI of patient 1 showed a large dural ectasia (*arrow*) in the midthoracic and lumbar spine (*C*). Abnormal bone structure and short and deformed long bones (*D*, humerus; *E*, radius and ulna; *F*, hand; *H*, tibia and fibula) in patient 1. Patient 2 had a pathologic fracture of the left femoral neck (*G*). Both patients had significant cranial hyperostosis (*I*, patient 1; *J*, patient 2).

The main features in the skeletal radiographs were (1) poor bone architecture—including abnormal bone size and shape and abnormal cortical and cancellous bone patterns—affecting all bones, (2) generalized degenerative arthritis (axial and appendicular) with loss of articular cartilage and intervertebral disks with fusion of joints with bone, and (3) extension of ossification from the bones into the soft tissues and interosseous membranes (Table 2). The radiographic findings were very similar in the two homozygote patients (Fig. 3), and both showed abnormal bone structure, short and deformed long bones, significant cranial hyperostosis, enthesopathies, and calcifications of the spinal ligaments in the cervical, thoracic, and lumbar spine (Fig. 3*A, B*); patient 2 had a pathologic fracture of the left femoral neck (Fig. 3*G*). Spinal MRI of patient 1 showed a large dural ectasia in the midthoracic and lumbar spine (Fig. 3*C*). The two heterozygous carriers of the mutation had no abnormalities on skeletal radiographs.

**Table 2:** Radiographic Characteristics of Skeletal Dysplasia Secondary to the Identified Homozygous *DMP1* Mutation

Skeletal site	Characteristic features
Skull	Thick calvarium and skull base
	Midface hypoplasia, prominent mandible
	Early loss of teeth
Cervial spine	Ankylosis of the cervical spine
	Disappearance of the intervertebral disks
	Posterior fusion of the spinous processes
	Ossification of the anterior longitudinal ligament
	Ossification of the laryngeal cartilages
Thoracic and lumbar spine	Tall vertebral bodies/vertebral compression
	Abnormal laminae and spinous processes
	Loss of disk space
	Ossification and extensive bony ankylosis
	Ossification of the paraspinal muscles and ligaments
	Scoliosis and kyphosis
Pelvis	Abnormal shape and bony architecture
	Loss of articular cartilage and bony ankylosis of the sacroiliac joints
	Deep acetabulae
	Degeneration of the hip joint space, osteophytes, ankylosis
Upper and lower limbs	Short, broad, and curved tubular bones
	Poorly developed cortices
	Calcification of the forearm interosseus membrane
	Ossification of tendon attachments
	Joint space narrowing with degenerative arthritis
	Pathologic lower limb fractures
Chest	Narrow chest
	Wide clavicles
	Degenerative arthritis of the shoulders and acromioclavicular joints

**Fig. 4:**
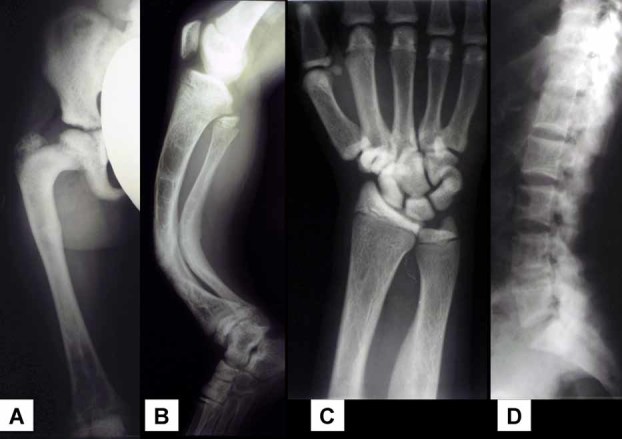
Radiographs of patient 1 obtained between 1952 and 1969. (*A*) Severe coxa vara (12 years) and (*B*) lower leg deformity (14 years). (*C*) Delayed closure of epiphyses and calcification of the forearm interosseus membrane (18 years). (*D*) Paraspinal calcifications without deformities of the vertebral column (29 years).

Review of earlier radiographs of patient 1 showed metaphyseal irregularities consistent with mild rickets, delayed bone maturation, and significant lower limb deformities (Fig. 4). Even at the age of 19 years, mild paraspinal calcifications were present.

Paraspinal calcifications (patients 1 and 2) and a prosthesis (patient 1) prevented BMD assessment in the homozygous individuals. One of the two heterozygote children showed reduced BMD suggestive of mild osteopenia (Table 1).

### Bone biopsy findings

**Fig. 5:**
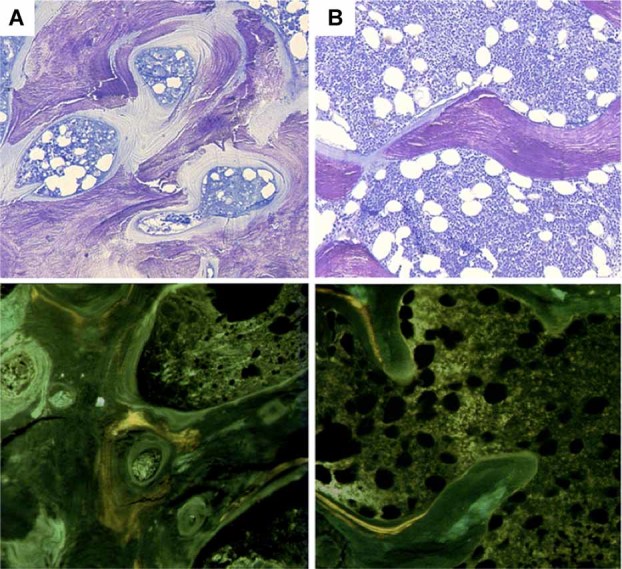
Bone histology, toluidine blue staining (*upper panel*). Dark-blue staining indicates mineralized bone; unmineralized osteoid stains pale blue. Osteoid accumulation was increased significantly throughout trabecular bone in patient 1(*A*), whereas focal areas of increased osteoid thickness were evident in individual 3(*B*). Tetracycline uptake (*lower panel*) is diffuse and abnormal in patient 1(*A*), whereas double and single labels are observed in individual 3(*B*).

Bone histomorphometry findings in patient 1 and her healthy son (individual 3) are presented in Table 3, and the histology is shown in Fig. 5. Patient 1, affected by ARHP, demonstrated very severe osteomalacia with marked increases in all osteoid parameters (ie, osteoid volume, surface, and thickness). No double-tetracycline labels were observed in this patient, and most of the single labels were broad and diffuse. The number of osteoblasts was within the normal range, but the number of osteoclasts was decreased. In contrast, individual 3, a heterozygous carrier of the *DMP1* mutation, displayed normal histomorphometric indices of osteoid accumulation. However, despite normal calculated bone indices, three to four focal areas of increased osteoid thickness were observed (Fig. 5). Tetracycline uptake was abnormal in patient 1, that is, broad and diffuse, and mineralization lag time (MLT) was increased, whereas measures of bone formation—mineralization apposition rate (MAR), mineralizing surface (MS/BS), and bone-formation rate (BFR)—were reduced compared with normal values. Osteoblast and osteoclast numbers also were decreased.

**Table 3:** Bone Histomorphometry Findings in Patient 1 (Female), Who Carries a Homozygous *DMP1* Mutation, and Her Son, Individual 3 (Male), Who Is Heterozygous for the Mutation

			Normal range	
			
Histomorphometric parameter	Subject 1	Subject 3	Female (61–70 years)	Male (31–40 years)
Bone volume/tissue volume (BV/TV) (%)	27.47	30.17	16 ± 4.2	22.0 ± 3.9
Osteoid volume/bone volume (OV/BV) (%)	57.7	1.86	1.5 ± 0.6	3.5 ± 1.9
Osteoid thickness (O.Th) (µm)	37.8	9.34	8.2 ± 3.7	9.7 ± 4.6
Osteoid surface/bone surface (OS/BS) (%)	84.55	14.16	13.1 ± 4.1	14.0 ± 4.6
Osteoblast surface/bone surface (Ob.S/BS) (%)	1.18	0.54	4.3 ± 1.8	6.0 ± 1.1
Eroded surface/bone surface (ES/BS) (%)	0	1.23	4.4 ± 2	4.5 ± 1.9
Osteoclast surface/bone surface (Oc.S/BS) (%)	0	0.264	0.8 ± 0.5	0.6 ± 0.4
Trabecular thickness (Tb.Th) (µm)	103.8	141.14	139.0 ± 38	138 ± 24
Trabecular separation (Tb.Sp) (µm)	273.98	326.73	602 ± 171	494 ± 82
Trabecular number (Tb.N) (/mm)	2.64	2.14	1.4 ± 0.4	1.7 ± 0.4
Mineralizing surface/bone surface (MS/BS) (%)	0.0001	2.9	7.2 ± 4.7	7.5 ± 3.6
Mineral apposition rate (MAR) (µm/day)	0.0001	0.45	0.68 ± 0.12	0.76 ± 0.18
Bone formation rate/bone surface (BFR/BS) (µm^3^/µm^2^/year)	0.0001	4.9	18 ± 3	20.8 ± 4
Adjusted apposition rate (Aj.AR) (µm/day)	0.0001	0.1	0.56 ± 0.21	0.63 ± 0.34
Mineralization lag time (MLT) (days)	**∞**	97	20.4 ± 8.1	18.4 ± 6.3

*Note:* Reference values given for each parameter as mean ± SD.

**Fig. 6:**
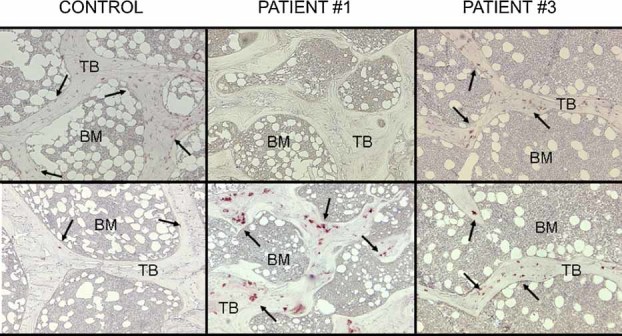
Immunohistochemical staining of DMP1 (*upper panel*) and FGF-23 (*lower panel*) expression in bone biopsy specimens of patient 1 (homozygote), individual 3 (heterozygote), and a normal control individual. DMP1 expression was absent in bone from patient 1 (A) whereas the bone specimen obtained from individual 3 (B) revealed DMP1 expression that is indistinguishable from that of controls. FGF-23 expression was increased in both subjects but to a greater degree in patient 1 (A) than in her son, individual 3 (B) TB = trabecular bone; BM = bone marrow.

Figure 6 displays the immunohistochemical expression of FGF-23 and DMP1. FGF-23 was increased in both patients; however, FGF-23 expression was markedly higher in the ARHP patient (patient 1) than in the heterozygous carrier of the *DMP1* mutation (individual 3). DMP1 protein was absent from bone of patient 1, and DMP1 expression in individual 3 was indistinguishable from that observed in controls.

## Discussion

We describe genetic and phenotypic findings in a not previously described family with autosomal recessive hypophosphatemia that is caused by a novel homozygous *DMP1* mutation. The clinical and laboratory observations in this family confirm that DMP1 has an important role in normal skeletal development and mineral homeostasis. Homozygous *DMP1* mutations result in hypophosphatemia owing to increased renal phosphate excretion and in a severe skeletal phenotype with short stature, deformed bones, abnormal bone structure, pathologic fractures, and severe enthesopathies. Furthermore, our findings suggest that *DMP1* mutation carriers also may have a clinically significant phenotype.

Previous studies have suggested multiple functions for DMP1 in postnatal skeletal development and chondrogenesis. DMP1 is expressed mainly in mineralizing tissues in hypertrophic chondrocytes, osteoblasts, and osteocytes and is required for normal growth plate and epiphyseal development.(5,6) Ablation of the *Dmp1* gene in mice results in a chondrodysplasia-like phenotype starting several days to weeks postnatally.(3,7) The growth plates are abnormally expanded and disorganized, and endochondral ossification is decreased. The metaphyses fail to lengthen normally, and the metaphyseal ends of long bones are wide and flared; also, the diaphyses of long bones are wide. Further, the epiphyseal formation and calcification are delayed.(7) These skeletal abnormalities appear to worsen progressively with age. The skeletal phenotype in our patients with ARHP was similar to that seen in Dmp1-deficient mice. The patients homozygous for the *DMP1* mutation had abnormal bone structure as well as short and deformed long bones. The skeletal abnormalities became apparent in early childhood and progressed with age, resulting in severe skeletal dysplasia with deformities and short stature. No radiographs were available from early childhood for the two homozygous carriers of the *DMP1* mutation. However, radiographs obtained between the ages of 12 and 19 years were consistent with delayed skeletal maturation and delayed closure of the growth plate in one of the patients.

In addition to the observed changes in the metaphyseal, epiphyseal, and diaphyseal areas of tubular bones, both patients had severe generalized degenerative arthritis (axial and appendicular) with loss of articular cartilage and intervertebral disk space. They also had significant cranial hyperostosis, enthesopathies, and calcifications of the spinal ligaments in the cervical, thoracic, and lumbar spine. The mechanism leading to the degenerative joint disease and ankylosis is unclear, but occurrence of these findings may indicate that DMP1 is required for normal long-term articular cartilage health. In fact, Ye and colleagues showed in mice that Dmp1 is essential for normal development of secondary ossification centers and that ablation of the *Dmp1* gene leads to delayed and malformed epiphyses followed by joint destruction.(7) Furthermore, altered architecture of the underlying subchondral bone may contribute to destruction of articular cartilage.(17) Ossification of the adjoining soft tissues occurred to an extent that has not been described previously in association with *DMP1* mutations and may be a reflection of the long-standing disease process. In contrast to the hypophosphatemia owing to an FGF-23-mediated increase in urinary phosphate excretion observed in patients lacking functional DMP1, *GALNT3* and *FGF23* mutations result in hyperphosphatemia owing to inappropriately low urinary phosphate excretion and low intact FGF-23 levels leading to the development of tumoral calcinosis. The severe enthesopathies observed in our patients are similar to those observed in other hypophosphatemic disorders, but these changes are usually much milder. Only one of our patients had been treated with phosphate, implying that the significant extraskeletal ossifications are not treatment-related but develop through another, still unknown mechanism.

Farrow and colleagues described a family with ARHP caused by a large biallelic deletion that removed most portions of the *DMP1* gene.(18) The two affected individuals in this study suffered from marked hypophosphatemia, persistent osteomalacia, stunted growth, nerve deafness, facial and dental abnormalities, and learning disabilities. Furthermore, Turan and colleagues(19) described a family with ARHP caused by a novel homozygous frameshift mutation (c.485Tdel; p.Glu163ArgfsX53) in exon 6 resulting in a premature stop codon. The affected children had, in addition to biochemical and radiographic findings typical for rickets, short and broad long bones and dysplastic distal phalanges. These skeletal changes are similar to but less severe than those observed in our patients. The profound skeletal abnormalities observed in our patients and the associated hearing impairment, as well as the dental anomalies, suggest that the phenotypic manifestations in our ARHP patients are significantly more severe than those observed in other forms of hypophosphatemic rickets.

One of the patients had a large dural ectasia, but it is unclear whether DMP1 is normally expressed in the dura mater and whether the lack of DMP1 in this tissue and/or hypophosphatemia contribute to the development of dural ectasia in ARHP. Dural ectasia occurs in genetic conditions with skeletal or connective tissue abnormalities, including Marfan syndrome, neurofibromatosis, and Ehlers-Danlos syndrome; in Marfan syndrome, dural ectasia associates with low BMD.(20,21) Dural ectasia also may occur in ankylosing spondylitis.(22) It has been proposed that the pulsatile changes in cerebrospinal fluid pressure lead to scalloping of the vertebrae, enlargement of the intervertebral foramina, and dural ectasia in a dural sac with abnormal elasticity and/or residual adhesions.(22)

The bones of *Dmp1*^−/+^ heterozygote mice were indistinguishable from those of wild-type littermates. Furthermore, both groups of animals showed comparable rates of weight gain and growth, as well as similar radiographic and histologic appearance, suggesting that the loss of a single copy of the *Dmp1* gene has no apparent effect on skeletal development.(8) Consistent with these findings in mice, the two heterozygous children of our ARHP patients showed normal stature, absence of skeletal deformities, and normal long bone and spinal radiographs. It remains unknown, however, whether heterozygous carriers of the *DMP1* mutation have an increased risk of developing early degenerative arthritis. Interestingly, both heterozygous individuals showed evidence of mild hypophosphatemia owing to increased urinary phosphate excretion. The calculated histologic indices of osteoid accumulation were normal when bone of heterozygous carrier 3 was evaluated, although this individual displayed evidence of focal osteomalacia, decreased number of osteoblasts, and an abnormal mineralization rate. Together these findings suggest a subtle mineralization defect, even in the heterozygous carriers, that may be related to a decrease in osteoblast number and/or osteoblast dysfunction. Consistent with a mineralization defect, bone FGF-23 expression appeared to be increased in this individual compared with bone samples from four healthy controls, whereas DMP1 expression was indistinguishable from that of controls. This suggested that the apparently normal amounts of DMP1 (ie, “inappropriately normal” amounts of the protein) were unable to suppress excess FGF-23 expression.

FGF-23, produced by osteocytes and osteoblasts, appears to be the key regulator of renal phosphate reabsorption and 1,25(OH)_2_D_3_ synthesis,(1) and increased circulating levels of this phosphaturic hormone are likely responsible for the biochemical and skeletal abnormalities observed in ARHP.(9) Osteoblastic bone formation modulates FGF-23 production, and regulation of FGF-23 is inversely linked to the production of bone matrix proteins, especially DMP1.(23) In *Dmp1*-null animals, hypophosphatemia and bone abnormalities can be normalized by transgenic expression of DMP1.(10) Furthermore, *Dmp1*-null mice with superimposed FGF-23 deficiency show no hypophosphatemia or 1,25(OH)_2_D_3_ deficiency and have normal skeletal mineralization.(9) It is therefore conceivable that, similar to other forms of hypophosphatemic rickets, administration of conventional phosphate–vitamin D analogue treatment during childhood could have partially corrected the skeletal phenotype in our patients with ARHP. Ideally, however, treatment should be targeted to FGF-23, potentially through the application of anti-FGF-23 antibodies, to mitigate the actions of endogenous FGF-23.(24)

In conclusion, the homozygous loss-of-function mutation in *DMP1* resulted in hypophosphatemia and severe progressive skeletal dysplasia characterized clinically by short stature, joint pain, contractures, and immobilization of the spine and radiographically by short and deformed long bones, significant cranial hyperostosis, enthesopathies, and calcifications of the paraspinal ligaments. Heterozygosity for the *DMP1* mutation was associated with mild hypophosphatemia but without clinical evidence of skeletal dysplasia. The skeletal phenotype in ARHP may be significantly more severe than that observed in other forms of hypophosphatemic rickets.
